# Supporting Measures to Improve Biosecurity within Italian Poultry Production

**DOI:** 10.3390/ani14121734

**Published:** 2024-06-08

**Authors:** Giuditta Tilli, Andrea Laconi, Francesco Galuppo, Guido Grilli, Artur Żbikowski, Arthi Amalraj, Alessandra Piccirillo

**Affiliations:** 1 Department of Comparative Biomedicine and Food Science, University of Padua, Viale dell’Università 16, 35020 Legnaro, Italy; giuditta.tilli@phd.unipd.it (G.T.); andrea.laconi@unipd.it (A.L.); 2 Unità Locale Socio-Sanitaria (ULSS) 6 Euganea, Via Enrico degli Scrovegni 14, 35131 Padua, Italy; francesco.galuppo@aulss6.veneto.it; 3 Department of Veterinary Medicine and Animal Science, University of Milan, Via dell’Università 6, 26900 Lodi, Italy; guido.grilli@unimi.it; 4 Department of Pathology and Veterinary Diagnostics, Institute of Veterinary Medicine, University of Life Sciences, Nowoursynowska 159C, 02-776 Warsaw, Poland; artur_zbikowski@sggw.edu.pl; 5 Department of Internal Medicine, Reproduction and Population Medicine, Faculty of Veterinary Medicine, Ghent University, Salisburylaan, 133, 9820 Merelbeke, Belgium; arthi.amalraj@ugent.be

**Keywords:** poultry, stakeholders, biosecurity, supporting measures, coaching, ADKAR^®^, Biocheck.Ugent^TM^

## Abstract

**Simple Summary:**

Biosecurity is essential for safeguarding the health and welfare of poultry flocks; however, its implementation may face many challenges. The challenges of implementing biosecurity measures in Italian poultry farms and the selection and validation of supporting measures (SMs) to improve compliance were addressed in this study. The methodology included stakeholder surveys, virtual farm tours, group discussions, and farmer coaching. While the level of biosecurity implementation was generally high, individual factors such as personality and behavior influenced its compliance. Virtual farm tours and group discussions proved effective in facilitating interaction and knowledge exchange among stakeholders. However, farmers’ preferences for minor changes due to time and cost constraints were highlighted during coaching sessions. Alternative methodologies like virtual farm tours and coaching have shown to be promising in engaging farmers and stakeholders, but further research on personal traits and attitudes is necessary to optimize compliance.

**Abstract:**

This paper describes the selection and validation of supporting measures (SMs) aimed at enhancing biosecurity compliance within Italian poultry farms. A tailored methodology, based on a stakeholders’ survey involving farmers and advisors, included a virtual farm tour, group discussion, and farmer coaching. Virtual farm tours and group discussions were delivered during two meetings targeting meat and egg production stakeholders, separately. Coaching was validated in 26 pilot farms (PFs) by assessing farmers’ attitudes towards change (i.e., ADKAR^®^) and farms’ biosecurity score (i.e., Biocheck.Ugent^TM^) before and after a minimum six-month period. A total of 20 out of 26 farmers agreed to implement at least one action plan (AP). Full implementation of the agreed APs was observed in ten farms, while others only partially implemented (*n* = 7) or did not implement (*n* = 3) the improvement. Most APs focused on enhancing house hygiene locks (*n* = 7), followed by bacterial auto-control after cleaning and disinfection (*n* = 4). Scoring tools indicated minimal or no variations in farmers’ attitudes towards change and farm biosecurity. Virtual farm tours and group discussions were found to be effective in fostering interaction and facilitating the exchange of experiences and knowledge among farmers and stakeholders of poultry production. Coaching indicated that farmers might prefer implementing minor changes possibly influenced by time and cost constraints associated with structural interventions. These limitations could have also impacted the scores of the farmer/farm. The findings of this study provide a foundation for further application of SMs to improve biosecurity in Italian poultry farms.

## 1. Introduction

On-farm biosecurity stands as the most effective tool to reduce the risk of introduction and dissemination of infectious diseases in and within livestock holdings [1] (World Organisation of Animal Health, 2023, https://www.woah.org/en/home/, accessed on 15 April 2024). It is a combination of practices encompassing three interconnected levels: conceptual, structural, and procedural [2]. Optimal on-farm biosecurity is achieved only when all three levels are addressed; focusing only on conceptual or structural improvements (e.g., disinfection arches, fencing, etc.) may be not sufficient. Since farm operators are responsible for implementing and complying with biosecurity practices on a daily basis, investment in procedural aspects (e.g., providing guidance, training, advising) is equally needed [3,4]. While conceptual and structural biosecurity are more easily verifiable and measurable because they are directly linked to their implementation, procedural biosecurity is more challenging due to its reliance on individual responsibility [5]. As highlighted in previous studies [3,6], assessing the implementation of procedural biosecurity practices (usually performed by interviewing farmers, farm workers, etc.) represents a critical point, since relying on factors like beliefs, attitudes, perceptions, education, background, and personality traits can influence the consistency of the individual’s response [7,8,9]. To ensure biosecurity compliance remains a priority; it is also crucial to acknowledge other potential barriers to implementation, including, for instance, limited time, financial constraints, and inadequate training [3,4,10].

Factors such as attitudes towards change among farmers and farm workers seem to significantly influence their willingness to implement biosecurity measures on the farms [11]. Therefore, when assessing biosecurity, it is essential to carefully consider such factors [6,9,12], as well as behavioral and psychosocial factors of the individual farmers’ behaviors and decision-making processes [13]. The attitude towards change, which involves recognizing the importance of making a change (i.e., understanding why a change is necessary or desirable), plays a pivotal role in achieving biosecurity goals. Among different behavioral models recognized, one for quantifying attitude towards change, known as ADKAR^®^ (Awareness, Desire, Knowledge, Ability, and Reinforcement), was developed by Hiatt (https://www.prosci.com/methodology/adkar, accessed on 15 April 2024) and later adapted to livestock farming by Houben et al. [14]. In addition to highlighting the importance of quantifying attitudes towards change, Houben et al. [14] emphasized the need to implement specific measures to support individuals through the change process. However, no adaptation of this tool in assessing the attitude towards change of poultry farmers on farm biosecurity has been previously developed, which has been achieved within the framework of the NetPoulSafe project (G.A. 101000728) [15].

In our context, the term “supporting measure” (SM) refers to a set of interventions (e.g., audits, trainings, videos, e-learning modules, etc.) aimed at improving the implementation and compliance with biosecurity in poultry production. While questionnaires [16] and checklists [17] are commonly used tools specifically designed for assessing on-farm biosecurity, they are often not readily accessible and available for consultation [9]. Therefore, efforts to find alternatives to improve biosecurity compliance in poultry holdings are needed [18,19]. Other SMs (e.g., coaching or participatory approach) exist but not all have been fully validated in the field, and their impact on biosecurity remains to be determined. For instance, the coaching of the farmer represents a form of guidance aiming at supporting the coachee (e.g., farmer) to find a long-term oriented solution to a specific challenge [20]. Recent studies have successfully validated coaching as a SM for antimicrobial stewardship in Belgium and the Netherlands [21]. Similarly, Ducrot et al. [22] adopted the participatory approach to reduce antimicrobial use in French pigs and poultry farms.

In Italy, the assessment of biosecurity implementation in poultry production primarily focuses on compliance with national legislation, and recent studies [3,17] have shown that compliance is high, mainly concerning the structural component. However, the current system of checks and questionnaires may have limitations, particularly regarding procedural biosecurity (i.e., procedures that cannot be evaluated at a given moment but rely on the statements provided by farmers/farm workers), which relies on the trustworthiness of the interviewed individual. To the best of the authors’ knowledge, no alternative methodologies have been explored or tested in Italian poultry production, nor has their impact on biosecurity improvements been evaluated. Therefore, the objectives of this study were to (a) identify and select the most successful and required SMs according to the Italian stakeholders’ perceptions; (b) implement the best/feasible/desirable/promising SMs in a network of pilot farms (PFs); (c) assess the effectiveness of the implemented SMs in the PFs; and (d) evaluate changes in biosecurity status in PFs and farmers’ attitudes towards biosecurity improvements.

## 2. Materials and Methods

### 2.1. Data Collection on Supporting Measures from Poultry Stakeholders

A questionnaire developed within the framework of the NetPoulSafe project was used to gather data on SMs aimed at improving biosecurity implementation. The questionnaire consisted of eight items, each containing a variable number of related SMs (i.e., “Biosecurity trainings” (*n* = 6); “Conducting information campaigns promoting biosecurity” (*n* = 5); “Educational material” (*n* = 4); “Biosecurity checks (audits)” (*n* = 2); “Regulations set up supporting biosecurity implementation” (*n* = 1); “Support by a biosecurity advisor (coach/vets)” (*n* = 3); “Organization of competition for best biosecurity (e.g., “biosecurity award”)” (*n* = 1); and “Financial support for biosecurity implementation” (*n* = 1). Additionally, a ninth item, “Other SMs (not described above)” was included to allow respondents to suggest SMs not listed in the questionnaire. Farmers and advisors could indicate for each SM whether it was already implemented on the farm (“successful” SM), whether it was needed (“required” SM), and whether each “successful” and/or “required” SM was effective, based on their opinion. Additionally, advisors could suggest the reasons for not implementing the required SMs and propose possible solutions on how to implement them. Questionnaires can be accessed in Files S1 and S2.

Sixty-seven randomly selected farmers and advisors, i.e., poultry companies’ veterinarians (*n* = 12), local public institutions (*n* = 6), academic experts *(n* = 5), other poultry veterinarians (*n* = 6), and producers’ organizations (*n* = 8)) were physically or virtually (i.e., video calls) interviewed between April and September 2021. Further details regarding the demographics of the interviewed farmers (*n* = 30) and advisors (*n* = 37) can be found in Laconi et al. [3]. Farms were situated in areas of significant importance for national poultry production, namely Veneto (*n* = 19), Lombardia (*n* = 5), Emilia Romagna (*n* = 3), Piemonte (*n* = 2), and Puglia (*n* = 1). The stakeholders represented various poultry sectors, including conventional broilers (*n* = 13) and layers (*n* = 13), free-range broilers (*n* = 8) and layers (*n* = 10), turkeys (*n* = 13), ducks (*n* = 3), and broiler breeders (*n* = 7).

### 2.2. Implementation and Validation of Supporting Measures in Pilot Farms

**Figure 1:**
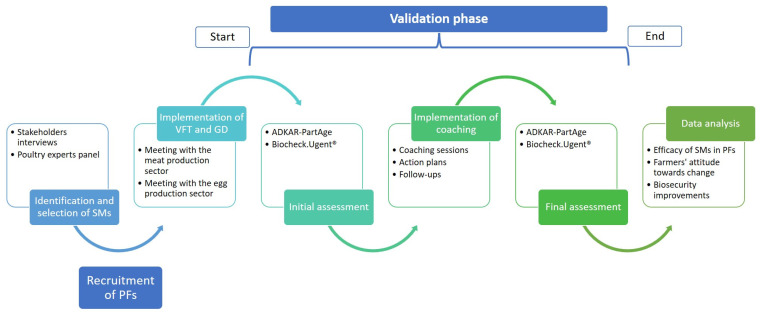
Schematic representation of the identification, selection, implementation, and validation steps of supporting measures in pilot farms.

Based on suggestions gathered from interviews with farmers and advisors, as well as inputs from a panel of experts (i.e., representatives from academia, official veterinary services, integrated companies, and farmers’ associations), the following SMs were identified as best/feasible/desirable/promising to be implemented and/or validated for improving biosecurity compliance in PFs: (1) virtual farm tour; (2) group discussion; and (3) coaching. Validation of the SMs was conducted only for coaching, following a predefined validation plan, while virtual farm tours and group discussions were implemented before the validation phase. The validation plan (Figure 1) included an initial assessment (using the ADKAR-PartAge and Biocheck. Ugent^TM^ (https://biocheckgent.com/en, accessed on 18 November 2023) tools to assess the farmer’s attitude towards change and the farm’s biosecurity score, respectively). Subsequently, coaching methodology and action plans (APs) were implemented and validated over a period of a minimum of six months. A final assessment, similar to the initial one, was conducted to detect any changes in both farmer’s attitudes and biosecurity scores resulting from the implementation of the SM. The validation phase started in May 2022 and concluded in July 2023.

#### 2.2.1. Supporting Measures

The purposes of the virtual farm tour and group discussion were to share biosecurity practices efficiently implemented on poultry farms with stakeholders and to gather feedback on biosecurity strengths and weaknesses, as well as farmers’ needs and preferences. Both were delivered through meetings involving farmers and other relevant stakeholders of the farms. These meetings were divided into two groups: one for conventional and free-range broilers, turkeys, and ducks (meat production sector), and another for conventional and free-range layers, and breeders (egg production sector). Each meeting started with a virtual farm tour, which consisted of screening a video comprising footage recorded in poultry farms showcasing various examples of biosecurity practices optimally implemented. Following the virtual farm tour, a group discussion was conducted to delve into the contents of the video. The contents of videos and group discussions were tailored according to the stakeholders’ sector (meat or egg). A moderator facilitated the discussion, and external experts and other stakeholders were also invited to stimulate discussion among farmers.

Coaching sessions aimed at developing an AP to improve biosecurity at the farm level by building a farm team made up of the farmer and the most relevant farm stakeholders. These sessions were designed to identify a specific, measurable, acceptable, realistic, and time-bound (SMART) plan to enhance on-farm biosecurity [23], following the principles of Plan–Do–Check–Act (PDCA) [24]. All coaching sessions were conducted by the same coach, one of the authors of the paper, who was adequately trained within the NetPoulSafe project. Each coaching session began with a discussion with the farmer (or other responsible individuals of the farm such as managers, technicians, etc.), along with other relevant stakeholders (farm team), to agree on an AP and define its implementation details. In cases of agreement, the farmer was given a six-month minimum period to implement the biosecurity change; during this period until the end of the validation phase, follow-up calls were carried out to monitor progress. The validation phase was considered concluded if no APs were agreed upon by the farm team.

#### 2.2.2. Pilot Farms

To implement and validate the selected SMs, a total of 29 PFs were recruited, covering various poultry categories: conventional broiler (*n* = 5) and layer (*n* = 4), free-range broiler (*n* = 5) and layer (*n* = 3), turkey (*n* = 6), duck (*n* = 3), and broiler breeder (*n* = 3) farms. Within the conventional layer category, both pullet (*n* = 2) and rearing phase (*n* = 2) were represented. Out of the 29 recruited PFs, 26 underwent the validation phase through coaching, as one free-range layer, one breeder, and one duck farm withdrew from the study. PFs were predominantly located in the Veneto region (*n* = 20, 76.9%), followed by Lombardia (*n* = 2, 7.7%), Emilia Romagna (*n* = 2, 7.7%), Piemonte (*n* = 1, 3.9%), and Puglia (*n* = 1, 3.9%).

#### 2.2.3. Assessment Tools

The ADKAR^®^ assessment tool, adapted to biosecurity based on the ADKAR^®^ model as described by Houben et al. [14] and Amalraj et al. [15], was used to measure farmers’ attitudes towards change. During the initial assessment of the validation phase, the first four blocks of the ADKAR^®^ model were explored, while the last block was examined only in the final assessment, following the implementation of the SM. Each building block was rated on a scale of 1 to 5, with a score of ≤3 indicating a potential obstacle to successful change. Additionally, the PartAge tool [10] was employed within the same questionnaire to gather additional information on participants’ age, gender, background, education, responsibilities, and satisfaction with work/life balance. The ad hoc ADKAR-PartAge questionnaire can be found in File S3. To assess any changes in biosecurity resulting from the implementation of the selected SM, the Biocheck. Ugent^TM^ scoring tool was administered either to the farmer or to the person in charge of the farm at the beginning and the end of the validation phase.

Each farmer or person responsible for the farm, participating in the study, was informed of the purpose of data collection and asked to sign an internal agreement. The internal agreement form can be found in File S4.

### 2.3. Data Analysis

The data were collected by using the ADKAR-PartAge questionnaires and the Biocheck. Ugent^TM^ assessment tool, extracted in Microsoft Excel^®^ 2019, and then analyzed by descriptive statistics. GraphPad Prism v10.1.2 was utilized for the analysis. To investigate differences between stakeholders’ opinions, the chi-square test with Yates’s correction was applied.

## 3. Results

### 3.1. Identification and Selection of Supporting Measures

**Figure 2:**
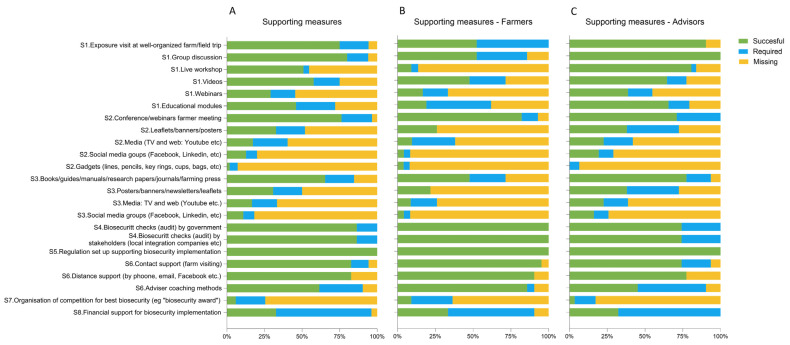
Successful and required supporting measures according to (**A**) farmers and advisors; (**B**) farmers; (**C**) advisors expressed as cumulative percentage (%).

The most successful SMs suggested by the stakeholders were as follows: regulations to set up supporting biosecurity implementation (100%; Item S5); biosecurity checks (audits) by government (86.4%; Item S4) or by stakeholders (e.g., integration companies) (86.4%; Item S4); direct (farm visiting) (82.7%) or distance support (by phone, email, etc.) (82.7%) by a biosecurity advisor (coach/vets) (Item S6); group discussions (80%; Item S1); conferences/webinars (76.3%; Item S1); exposure visits at well-organized farms/field trips (75%; Item S1); books/guides/manuals/research papers/journals/farming press (65.4%; Item S3); and farm coaching methods (61.5%; Item S6) (Figure 2A). Stakeholders’ opinions (Figure 2B,C) varied in terms of group discussions (*p* = 0.0001), live workshops (*p* < 0.0001), educational modules (*p* = 0.003), and books/guides/manuals/research papers/journals/farming press (*p* = 0.0002), which were primarily deemed successful by the advisors, while biosecurity checks (audits) by government (*p* = 0.0121) or by stakeholders (*p* = 0.0121), and farm coaching methods (*p* = 0.0078) by the farmers. Despite SMs providing an incentive for improvement, such as financial support (63.5%; Item S8) and organization of competitions for the best biosecurity (e.g., “biosecurity award”) (19.6%; Item S7), being the most required SMs as suggested by the stakeholders (Figure 2A), SMs providing direct support and training/information/education, such as coaching (28.8%; Item S6), educational modules (26%; Item S1), media (TV and web: YouTube, etc.) (23.1%; Item S2), exposure visits at well-organized farm/field trips (19.2%; Item S1), leaflets/banners/posters (19.2%; Item S2), books/guides/manuals/research papers/journals/farming press (19.2%; Item S3), posters/banners/newsletters/leaflets (19.2%; Item S3), videos (17.3%; Item S1), media: TV and web (YouTube, etc.) (16.7%; Item S3), conferences/webinars (16.4%; Item S2), and group discussions (14%; Item S1), were also considered. Live workshops (*p* = 0.0033) were indicated as required only by the farmers, while leaflets/banners/posters (*p* = 0.0054) and posters/banners/newsletters/leaflets (*p* = 0.0054) were mentioned only by the advisors (Figure 2B,C). Stakeholders’ opinions according to the different poultry sectors are detailed in File S5. No statistical analyses were performed due to the low number of individuals interviewed for each sector.

### 3.2. Implementation and Validation of Supporting Measures

The virtual farm tour and group discussion were addressed during two meetings involving both the meat and egg production sectors. In the meat production sector meeting, the majority of participants were farmers (*n* = 12, 52.2%), with half of them (*n* = 6, 50%) belonging to the turkey production (Table 1 and File S6). Conversely, during the egg production sector meeting, the majority of participants were stakeholders rather than farmers (*n* = 11, 84.6% vs. *n* = 2, 15.4%); no representatives from the breeder farms participated in the meeting (Table 1 and File S6).

Out of the 29 recruited PFs, 26 underwent the validation phase of coaching, as three farmers did not participate either because they were not motivated enough (*n* = 1) or because of Avian Influenza (AI) outbreaks (*n* = 2). The participants targeted by the coaching sessions included farmers (*n* = 21, 80.8%), farm technicians (*n* = 2, 7.7%), farm managers (*n* = 1, 3.9%), farm workers (*n* = 1, 3.9%), and farm veterinarians (*n* = 1, 3.9%) (Table 1 and File S6). Other relevant stakeholders (range 1 to 5), such as official and/or farm veterinarians, farm technicians, farm workers, integrated company representatives, or external experts, also participated as part of the farm team (File S6). During each coaching session, critical points were identified and APs were discussed and, if agreed upon, implemented. Subsequently, follow-up calls were conducted according to the PDCA approach. In Table 2, the durations of the validation phase, coaching sessions, and follow-up calls are summarized.

Out of 26 PFs recruited in the validation phase, the majority of them (*n* = 20, 76.9%) agreed to implement at least one AP, resulting in a total of 22 APs (range one to three) being agreed upon (Table 3). Regarding the remaining six farms, two of them (7.7%) did not agree to a specific plan, but the farmers expressed interest in receiving specific infographics on external crews for the farm. Concerning the other four farms (15.4%), it was not possible to reach an agreement on any AP. Among this group, two out of the four farms ceased their activity during the validation period, with one farm closing down and the other being affected by a natural calamity (i.e., flooding). Most of the APs were fully implemented during the validation phase (*n* = 12, 54.6%), while some were started but not finished (*n* = 7, 31.8%), or, despite an initially agreed plan, not implemented (*n* = 3, 13.6%). Out of the 22 agreed-upon plans during the coaching sessions, farmers rarely suggested any AP (*n* = 2, 9.1%). In most cases, other members of the farm team proposed potential intervention measures, with official veterinarians suggesting APs in nine cases (40.9%) and integrated company representatives (veterinarians or technicians) in ten (45.5%). In one instance (4.5%), an external expert/coach suggested an AP. Out of the 20 PFs that agreed to an AP, fourteen farms (70%) required follow-up calls to monitor the implementation phase. APs comprised eleven different interventions; among them, improvements of house hygiene locks (*n* = 7, 31.8%) and microbiological screening after cleaning and disinfection procedures (*n* = 4, 18.1%) were the most frequently agreed upon.

In four out of 26 PFs recruited for the validation phase, the ADKAR-PartAge questionnaire was filled by two individuals from the same farm. Additionally, for two PFs, the farmer/farm veterinarian did not complete the validation phase. Therefore, these six PFs were excluded from the analysis. The majority of individuals interviewed using the PartAge questionnaire (File S6) were male (*n* = 15, 75%), aged between 35 and 50 years old (*n* = 10, 50%). Their main background was being a poultry farmer, either lifelong (*n* = 8, 40%) or after transitioning from a different profession (*n* = 5, 25%). The most common education level attained was up to high school (*n* = 14, 70%), and all reported being rather satisfied with their current position. Additional information on biosecurity training received was not collected; however, integrated companies usually provide farmers with periodic trainings on biosecurity. Between the initial and final assessment of the validation phase, no changes in the average scores of the first four ADKAR^®^ blocks were observed (Table 4 and File S6). However, all blocks scored higher than three, indicating no potential barriers to change.

In summary, farmers demonstrated moderate to full awareness of the positive effects of improved biosecurity on health and productivity, potentially indicating their willingness to improve biosecurity on their farms (Awareness); a desire to improve biosecurity to the extent possible, considering the potential costs involved (Desire); knowledge of the biosecurity measures to be implemented, indicating their readiness to draft an AP (Knowledge); and ability, either partially or fully, to invest money and/or time to implement the AP (Ability). The fifth block (Reinforcement) was only measured during the final assessment and scored 3.2 on average, suggesting that farmers were neither negatively nor positively impacted by the change. This could be explained by the fact that the APs implemented did not result in significant or tangible improvements in animal performances, economic benefits, or work satisfaction during the relatively short observation period. Reinforcement is reported to be a decisive factor in convincing farmers of the importance of biosecurity as it represents an attempt to quantify the biosecurity effects. Similarly, the Biocheck.Ugent^TM^ scores slightly increased only in conventional and free-range broiler farms (Table 5 and File S6).

No changes were observed in the scores for all the other poultry categories, except for duck farms where the score decreased. This happened because during the validation phase, there were some AI outbreaks that severely impacted the original day-old-ducks supplier, and the farmers (under the same integration) were obliged to change suppliers, which also delayed yearly production. On average, the internal biosecurity score was slightly higher than the external one (85.2 vs. 80.8 at T0 and 85.3 vs. 80.7 at T1, respectively). While an increase in the internal biosecurity score (83.0 vs. 84.0) was observed in duck farms, a slight decrease in the external one (78.5 vs. 76.5) was noted at the end of the validation phase.

## 4. Discussion

Stakeholders’ and experts’ opinions served as a valuable starting point for identifying and selecting the best/feasible/desirable/promising SMs to implement and/or validate in PFs. Regulations and audits by government or stakeholders regarding biosecurity implementation were identified as highly successful by both stakeholders. This is likely because of the strict legislation in Italy and the integration of poultry production [3,17], leading to regular enforcement of biosecurity measures. However, while legal enforcement (i.e., by law or audits) ensures compliance because farmers are obliged to be compliant, it may not necessarily enhance either farmer’s awareness of the importance or willingness for the improvement of biosecurity [25]. Interestingly, financial support emerged as the most required SM by both farmers and advisors. However, it was beyond the scope of the project to provide direct funding for biosecurity improvement to the recruited PFs. Nonetheless, it is important to acknowledge the economic constraints faced by farmers, particularly in implementing structural biosecurity measures that may require substantial investment [4]. In agreement with previous observations [3], this finding indicates a strong awareness among poultry stakeholders regarding the importance of biosecurity and the necessity for financial assistance from national and/or European institutions/governing bodies to facilitate biosecurity improvements. Several SMs related to training (e.g., exposure visits at well-organized farm/field trips, group discussions), information (e.g., conferences/webinars), and education (e.g., books/guides/manuals/research papers/journals/farming press) were deemed successful and/or required by stakeholders for improving biosecurity compliance. This suggests that poultry stakeholders recognize the significance of improving knowledge and awareness of biosecurity through traditional methods, while also being open to innovative approaches that promote interaction among all stakeholders in the poultry sector [22]. Similarly, stakeholders emphasized the need for direct support from specialized trainers or coaches to ensure the correct application of biosecurity practices, as reported also by Cui and Ping Liu [18] and Scott et al. [19]. Therefore, virtual farm tours, group discussions, and farmer coaching were selected as SMs to test in the PFs network. To enhance stakeholders’ knowledge and awareness of biosecurity, a virtual farm tour (preferred over “exposure visits at well-organized farm/field trips” because it is considered to be more “biosecure”) and group discussion were selected, because capturing the perception that they preferred to use/be involved in practical and interactive activities. Coaching was chosen because it is a methodology that helps farmers understand the benefits of biosecurity through non-directive questioning and interaction [21].

The combination of virtual farm tours and group discussions during the same event seemed to have been well received by farmers and the other stakeholders attending the meetings since it fulfilled the primary needs of identifying practical solutions to common challenges while also communicating and sharing biosecurity experiences. Virtual farm tours represented a valid alternative to physically visiting well-organized poultry farms, as well as exploring different poultry farm settings to show optimal implementation of biosecurity practices. At the same time, it served as a compromise to comply with one of the pillars of biosecurity, namely preventing the entry of multiple individuals into a poultry farm [26]. By avoiding this, it contributed to reducing the risk of introducing and spreading infectious agents. This approach should be promoted in poultry production, as it represents a step forward in selecting alternatives to support biosecurity interventions, leveraging technology for improvement, as highlighted by Racicot et al. [6]. Furthermore, group discussion seemed to have a positive impact on farmers in terms of interaction and sharing information [27]. Despite the vertically integrated poultry production systems, which may limit information sharing, the purpose of these discussions was not to compare but to foster knowledge exchange among peers in the poultry sector. Such a methodology proved to be beneficial as it encouraged communication among stakeholders with similar experiences. This shift in approach, from promoting implementation through directives to promoting implementation through discussing and listening, has been highlighted as a critical point in previous studies [28].

In contrast to group discussion, which collectively facilitated the identification of critical points in poultry production, coaching enabled us to achieve this goal individually at the farm/farmer level. This methodology relies on communication techniques aimed at encouraging the farmer to find improvements independently, as previously described in other studies [21,29]. Indeed, the difference between coaching and advising lies in allowing the farmer to achieve long-term oriented solutions by identifying a specific intervention plan with the support of the farm team in the former, while in the latter, farmers passively receive information from stakeholders. However, in our coaching sessions, the participation of different stakeholders in the farm team proved to be important for helping the farmer find an improvement strategy. In our study, both decision-making and investment processes were suggested mainly by official veterinarians or representatives of integrated companies rather than by the farmers themselves. Indeed, in some cases (four farms), both the decision to implement the AP, i.e., microbiological screening after cleaning and disinfection procedures, and its associated cost of implementation were supported by the integrated company. The majority of APs consisted of minor improvements, suggesting that farmers may be inclined to implement small changes, either because they believed they had already implemented all required measures or because they perceived the implementation process as time-consuming and costly, which has been reported as a blocking factor [3,4]. Moreover, in our study, the short duration of the validation phase may have also hampered more substantial interventions due to time restrictions. This also impacted some APs that were either partially implemented or not implemented, e.g., fencing surrounding the farm or structural interventions inside the poultry house. Nevertheless, less challenging structural improvements, such as the bench of the house hygiene lock, were implemented. Although seemingly minor, these interventions hold significant importance, as they represent farmers’ willingness towards change. This also suggests that improving on-farm biosecurity can be achieved through continuous and routine daily efforts. It is worth noting that for coaching to be successful, it is imperative that farmers identify solutions themselves with the support of the coach [20]. Our findings suggest that the effectiveness of coaching may depend not only on the personality of the farmer [14] but also on the context of the poultry production system, whether integrated or not. Indeed, minimal or no changes might also be an indication of the vertically integrated system, which provides constant support to each farmer belonging to a specific company. Our findings suggest that the methodology should be adjusted considering the integration of poultry production. Consequently, coaching the personnel of the integrated company together with the farmers may be more effective. In our study, coaching sessions facilitated discussion among diverse members of the farm team, who generally work together but seldom have the opportunity to collectively discuss improvement plans with the farmer. However, these discussions were not always straightforward, leading to disagreements in finding APs due to different priorities of each participant. In this context, the role of the coach was crucial in guiding the discussion and focusing the attention on the ultimate goal [20]. In our coaching sessions, successful APs often resulted from strong, trusting relationships between parties, while a lack of trust led to unsuccessful ones. Generally, the nature of the relationship between the farm veterinarian and the farmer influenced the outcome of the coaching session. For instance, a positive relationship facilitated the implementation of the veterinarian’s suggested plan without further discussion (e.g., the coating of surfaces in the hygiene lock, which can be costly). This likely reflects the trust built over years among parties. Conversely, when trust was lacking, coaching sessions did not always have a positive outcome and sometimes exacerbated existing disagreements. Our findings suggest that interpersonal relationships are another important factor influencing the effectiveness of this methodology and should be considered when planning the activity.

The interviews conducted to gather farmers’ personal information and assess their attitudes towards change were not always straightforward. However, farmers exhibited no particular barriers to change, suggesting possible opportunities for improvement. Some farmers were hesitant to provide information, particularly regarding their education and background, probably related to their personality traits. Only one study [15] has been conducted on the use of this tool for measuring farmers’ attitudes towards change in biosecurity, which showed significant changes in three (“Awareness”, “Desire”, and “Knowledge”) of the four attitude elements in 155 poultry farmers of seven European countries. Similarly, previous research conducted by Houben et al. [14] and Caekebeke et al. [21] reported differences in attitudes towards change in different countries, which could be related to the policy (presence or absence of recent awareness campaigns) of the countries or different perceptions of the stakeholders. However, changes in the ADKAR^®^ elements after the intervention were noted. Even though no changes were detected in the “Awareness”, “Desire”, “Knowledge”, and “Ability” among farmers in our study, this could be attributed to the short period of the validation phase, which also impacted the measurement of “Reinforcement”. Therefore, longer observation periods are strongly recommended, considering also that some measures, especially those that are structural, require time and financial investments for implementation. Although a slight increase in biosecurity scores was observed in conventional and free-range broilers, the scores were consistently above 80% for all poultry categories (except for duck farms), indicating an overall high level of implementation. This aligns with findings from national checklists [17] and perceptions from farmers and advisors [3], confirming a high level of biosecurity in Italian poultry production. Only slight changes in the Biocheck.Ugent^TM^ scores between the initial and final assessment were detected probably due to the fact that the majority of APs consisted of minor improvements, which were not captured in the tool due to the lack of the relative question. Therefore, these findings may underestimate the true impact of implemented measures.

## 5. Conclusions

Biosecurity is widely recognized as crucial in poultry farming; however, its compliance can pose significant challenges. The high level of biosecurity observed in this study reflects a strong awareness among Italian farmers and stakeholders. However, individuals themselves play a vital role in determining compliance, influenced by factors like personality and behavior. This study explored alternative SMs such as virtual farm tours, group discussions, and coaching tailored to farmers and stakeholders to improve biosecurity compliance. Many farmers showed considerable engagement throughout the entire process, from the initial data collection to the validation phase, indicating an appreciation for these methodologies and a willingness to improve biosecurity. Hence, alternative methodologies directed at farmers seem to hold promising results and offer benefits for enhancing biosecurity. However, further research on personal traits and attitudes towards biosecurity is needed to achieve optimal compliance in Italian poultry farms.

## Figures and Tables

**Table 1:** Poultry stakeholders and sectors involved in the implementation and validation of supporting measures (i.e., virtual farm tours, group discussions, and coaching).

	Virtual Farm Tour and Group Discussion		Coaching *
	Meat Production Sector Meeting	Egg Production Sector Meeting	
	No. (%)	No. (%)	No. (%)
Stakeholders involved in the implementation and validation of supporting measures			
Farmer	12 (52.2)	2 (15.4)	21 (80.8)
Farm manager	1 (4.3)	2 (15.4)	1 (3.9)
Farm worker	4 (17.4)	-	1 (3.9)
Farm technician	-	1 (7.7)	2 (7.8)
Farm veterinarian	-	2 (15.4)	1 (3.9)
Official veterinarian	1 (4.3)	1 (7.7)	-
External expert (public institution, research)	5 (21.8)	5 (38.5)	-
Total	23	13	26
Poultry production sectors in the implementation and validation of supporting measures			
Broilers	5 (29.4)	-	5 (19.2)
Free-range broilers	4 (23.5)	-	4 (15.4)
Layers	-	4 (57.1)	4 (15.4)
Free-range layers	-	3 (42.9)	3 11.5)
Turkeys	6 (35.3)	-	6 (23.1)
Ducks	2 (11.8)	-	2 (7.7)
Breeders	-	0	2 (7.7)
Total	17	7	26

* Only the coached person is reported.

**Table 2:** Duration of the validation phase, coaching sessions, and follow-up calls to farmers and/or other relevant stakeholders according to the poultry production category. Means and standard deviations (within brackets) are reported.

	Validation Phase (Days)		Coaching Session (min.)		Follow-Up Calls (min.)	
	No.	Mean (Standard Dev.)	No.	Mean (Standard Dev.)	No.	Mean (Standard Dev.)
Conventional layers	4	301 (32.1)	4	67.5 (15)	1	3.5
Free-range layers	2	242 (42.4)	3	50 (17.3)	1	NA
Conventional broilers	5	270 (32.3)	5	63 (16.4)	3	4.2 (1.4)
Free-range broilers	4	302 (20.4)	4	78.8 (28.4)	4	4.6 (1.7)
Breeders	2	262 (34.7)	2	60	0	NA
Turkeys	5	250 (28.7)	6	62.5 (6.1)	3	3.9 (4)
Ducks	2	296 (24)	2	75	2	9
Total	24	275 (34.6)	26	65.2 (16.4)	14	4.7 (2.8)

**Table 3:** Summary of the action plans implemented in pilot farms during the validation phase.

	Pilot Farms	
	No.	%
Action plans agreed to by the farm team during the coaching session		
No implementation of any AP in the farm	6	23.1
Implementation of one AP in the farm	19	73.1
Implementation of more than one AP in the farm (i.e., 3)	1	3.8
Total	26	100
Status of the action plans’ implementation during the validation phase		
Farms fully implementing the AP	12	54.6
Farms partly implementing the AP (i.e., started but not finished)	7	31.8
Farms that failed to implement the agreed AP (i.e., never started)	3	13.6
Total	22 *	100
Member of the farm team suggesting the action plan		
Farmer	2	9.1
Integrated company representative (technician, veterinarian)	10	45.5
Coach/external expert	1	4.5
Official veterinarian	9	40.9
Total	22 *	100
Member of the farm team contributing to the investment in the action plan		
Integrated company representative	4	18.2
Farmer	17	77.3
Others	1	4.5
Total	22 *	100
List of agreed practices/measures		
Direct support by a stakeholder	1	4.6
Fencing of the farm area	1	4.6
Bacterial auto-control after cleaning and disinfection (C&D)	4	18.1
Signages/banners in the farm area	1	4.6
Improvements in the house hygiene lock	7	31.7
External dissuasive systems for wild animals	2	9
Dedicated footwear (boots) and disposable clothes for catching crews	2	9
Improvements in the brooding area	1	4.6
Internal improvements of the poultry house (i.e., wall cracks)	1	4.6
Increased no. of dedicated footwear per poultry house	1	4.6
Improvements in litter management	1	4.6
Total	22 *	100

* Three plans were agreed on in the same farm.

**Table 4:** ADKAR^®^ profiles of the farmers/farm responsible persons involved in the validation phase. Mean scores and standard deviations (within brackets) are reported according to the poultry production category and the results of the initial (T0) and the final (T1) assessment.

	No. of Farms	A		D		K		A		R	
		T0	T1	T0	T1	T0	T1	T0	T1	T0	T1
Breeders	1	4	4	4	4	4	4	4	4	NA	3
Broilers	3	4	4	4.33 (1.15)	4.33 (1.15)	3.67 (0.68)	3.67 (0.68)	4.67 (0.58)	4.67 (0.58)	NA	3
Free-range broilers	4	4.25 (0.5)	4.25 (0.5)	4.75 (0.5)	4.75 (0.5)	4.5 (0.58)	4.5 (0.58)	4.25 (0.5)	4.25 (0.5)	NA	3.75 (0.96)
Layers	4	5	5	4.75 (0.5)	4.75 (0.5)	3.5 (1)	3.5 (1)	4 (0.82)	4 (0.82)	NA	3
Free-range layers	2	4.5 (0.71)	4.5 (0.71)	5	5	4.5 (0.71)	4.5 (0.71)	5	5	NA	3
Turkeys	4	4.25 (0.5)	4.25 (0.5)	4. 5 (0.58)	4. 5 (0.58)	4. 5 (0.58)	4. 5 (0.58)	4. 5 (0.58)	4. 5 (0.58)	NA	3.25 (0.5)
Ducks	2	4	4	3.5 (0.71)	3.5 (0.71)	3.5 (0.71)	3.5 (0.71)	2.5 (0.71)	3.5 (0.71)	NA	3
Total	20	4.35 (0.71)	4.35 (0.71)	4.5 (0.69)	4.5 (0.69)	4.05 (0.76)	4.05 (0.76)	4.2 (0.83)	4.2 (0.83)	NA	3.20 (0.52)

**Table 5:** Biocheck.Ugent^TM^ scores of pilot farms involved in the validation phase. Mean scores and standard deviations (within brackets) are reported according to the poultry production category and the results of the initial (T0) and the final (T1) assessment.

	No. of Farms	Average Biosecurity Scoring		External Biosecurity Scoring		Internal Biosecurity Scoring	
		T0	T1	T0	T1	T0	T1
Breeders	1	83	83	83	83	83	83
Broilers	5	81.60 (5.08)	82 (5)	81 (6.44)	81.4 (6.43)	83 (4.53)	83 (4.53)
Free-range broilers	4	81.5 (6.86)	81.75 (6.75)	78.25 (7.41)	78.25 (7.41)	89.75 (5.91)	90 (6.06)
Layers	3	82.33 (9.07)	82.33 (9.07)	81.33 (7.51)	81.33 (7.51)	83 (11.36)	83 (11.36)
Free-range layers	2	84 (4.24)	84 (4.24)	84 (5.66)	84 (5.66)	82.5 (0.71)	82.5 (0.71)
Turkeys	4	83.75 (4.79)	83.75 (4.79)	81.75 (5.19)	81.75 (5.19)	88 (5.23)	88 (5.23)
Ducks	2	80 (4.24)	79 (4.24)	78.5 (2.12)	76.5 (2.12)	83 (9.9)	84 (9.9)
Total	21	82.23 (5.21)	82.29 (5.22)	80.81 (5.6)	80.71 (5.72)	85.19 (6.35)	85.33 (6.38)

## Data Availability

Some of the data presented in this study are available upon request from the corresponding author. The data are not publicly available due to privacy concerns.

## Supplementary Materials

The following supporting information can be downloaded at: https://www.mdpi.com/article/10.3390/ani14121734/s1, File S1: questionnaire used to collect information from Italian poultry farmers on the implementation of biosecurity measures; File S2: questionnaire used to collect information from Italian poultry advisors on the implementation of biosecurity measures; File S3: PartAge-ADKAR questionnaire used to collect information from Italia poultry stakeholders; File S4: internal agreement form; File S5: successful and required supporting measures according to stakeholders of different poultry sectors. File S6: Characteristics of the pilot farms involved in the implementation and validation of SMs.
