# Supplementary material for: Supporting Measures to Improve Biosecurity within Italian Poultry Production
Source: Animals (Basel). 2024 Jun 8;14(12):1734. doi: 10.3390/ani14121734 (PMC11201041; doi:10.3390/ani14121734)
Supplement: Supplementary file 1 [file animals-14-01734-s001.zip › File S3_ADKAR-Partage.pdf]

PARTAGE QUESTIONNAIRE

PILOT FARM

CODE________________ **DATE**________________

**Age**	<35 years old<br>35-50 years old<br>>50 years old
**Gender**	Male<br>Female
**Background**[1]	
**Education**[2]	
**Who does what?**[3]	
**Satisfaction work/life balance**	Quite satisfied<br>Rather not satisfied

[1] Fill in all the information you think is relevant to describe the farmer's career path, in particular if his family comes from the farming world, if the person has had other jobs, other farms, changed production or not during his career.

[2] Fill in the highest level of diploma obtained by the person and specify if it is related to his current activity as a breeder. If not, specify what agricultural training the person has.

[3] Try to describe with the farmer how the work is organized on the farm, especially if there are employees, who takes care of what, who is responsible for what.

ADKAR QUESTIONNAIRE

PILOT FARM

CODE________________ DATE________________

NetPoulSafe

	Score	Description
**Awareness**	1	Farmer missed all information regarding biosecurity. Is not aware that improving biosecurity results in reduced risk of introduction and spread of infectious diseases/pathogens.
	2	Farmer is aware of the recommendation to improve biosecurity, but is completely denying the potential effects of better biosecurity on the risk of infectious disease introduction and spread.
	3	Farmer is aware that biosecurity should be improved, but contests the effect on animal health and production. Mentions that disease introduction cannot be avoided anyway.
	4	Farmer is aware that biosecurity should be improved and positive effects on health and productivity of the flock are expected and accepts that some changes are required to achieve this.
	5	Farmer is fully aware that biosecurity should be improved. He takes responsibility for the biosecurity in the farm and embraces the required improvement for the farm.
**Desire**	1	Farmer states: “This is not my problem. It does not concern me”.
	2	Farmer will improve, but is not the first adaptor. Farmer states: “my “neighbour” should also improve”.
	3	Farmer wants to improve, but slowly. The goal is not to become the farm with the best possible biosecurity, just enough is also OK.
	4	Farmers goal is to improve the biosecurity as much as possible, yet without substantial costs.
	5	Farmers goal is to improve the biosecurity as much as possible, even if there are considerable costs related to this improvement.
**Knowledge**	1	It is not clear what the risks for introduction and spread of infection on the farm are. It is not possible to draw up an action plan. The farmer and his network really do not know where to start.
	2	Either it is not known / understood which biosecurity improvements are required or there is low or inaccurate knowledge, experience or skills with regard to the execution of the biosecurity improvements.
	3	Information on the infection introduction risks are available and clear for the farmer, action plan can be drawn up.
	4	Information is available and clear, but some discussion about the implementation is still present. Support for the farm and farmer is needed to implement change.
	5	Information is available and clear; action plan is accepted and knowledge and skills are sufficiently available at level of farmer and his network.
**Ability**	1	Farmers sees only obstacles for every proposed change and therefor does not implement any.
	2	Farmer implements a limited number of changes which are easy to achieve. The selection is not made upon expected effect, but on requested input.
	3	Some changes are accepted and implemented in the farm. Or implementation is saved for the rebuilding or new building.
	4	Farmer is implementing systematically. But money or time are hampering some changes.
	5	Farmer is investing time, money and/or effort to implement changes.
**Reinforcement**	1	Farmer has negative experiences with improving biosecurity.
	2	Farmer received or receives negative feedback from the personal environment with regard to changed biosecurity measures.
	3	Improved biosecurity is not perceived to have positive or negative effects.
	4	Improved biosecurity has led to more job satisfaction and better herd performance.
	5	Improved biosecurity has led to better economic performance or a higher personal status.

NOTE: The “Reinforcement” component will not be scored at this moment as no supporting measures are implemented yet.

1. lowest score
5. highest score

Each element with score of 3 or less blocks change.
