# Supplementary material for: Supporting Measures to Improve Biosecurity within Italian Poultry Production
Source: Animals (Basel). 2024 Jun 8;14(12):1734. doi: 10.3390/ani14121734 (PMC11201041; doi:10.3390/ani14121734)
Supplement: Supplementary file 1 [file animals-14-01734-s001.zip › File S4_NetPoulSafe_EngagementLetterAddendum_ENG.pdf]

NetPoulSafe

Informed consent

I, the undersigned …………………………………………………………………(surname & first name) hereby confirm that I was informed about the project "NETPOULSAFE”.

The researcher has given me sufficient information with regard to the purpose and design of the project, the conditions and duration thereof. Moreover, I was given sufficient time to think and to ask questions, to which I received satisfactory answers. I understand that I can stop my participation in this study at any time. I authorize the project manager to collect, process and use my information. Discussions remain within the AKIS network, only joint conclusions are shared with my identity secret. If I share confidential information orally, I will indicate this.

“I agree to participate in the “NETPOULSAFE” project as : Pilot farm actor

- ☐ As a pilot farm actor, I participate in this validation process. I will do my best to apply the measures as much as possible. I agree to participate in company-level interviews and data collection. I also agree that the researchers will make an assessment of my attitude towards biosecurity.
- ☐ I give permission to take pictures of my company
- ☐ I give permission to take pictures of poultry on my farm

Signature participant: Date…………………………………….

Project manager
I hereby declare, the undersigned ………………………………………………… (surname and first name) that I have given the volunteer all necessary information in connection with ‘NETPOULSAFE' and as well as a copy of the document 'information sheet for participants'. I confirm that no pressure was exerted on the volunteer to participate in this study. I also confirm that I am willing to answer any additional questions that may arise.

Signature researcher: Date…………………………………..

If you have any questions you can always contact [xxxxx].
Thank you in advance for your participation!
