# Supplementary material for: Supporting Measures to Improve Biosecurity within Italian Poultry Production
Source: Animals (Basel). 2024 Jun 8;14(12):1734. doi: 10.3390/ani14121734 (PMC11201041; doi:10.3390/ani14121734)
Supplement: Supplementary file 1 [file animals-14-01734-s001.zip › File S5.pptx]

## Slide 1
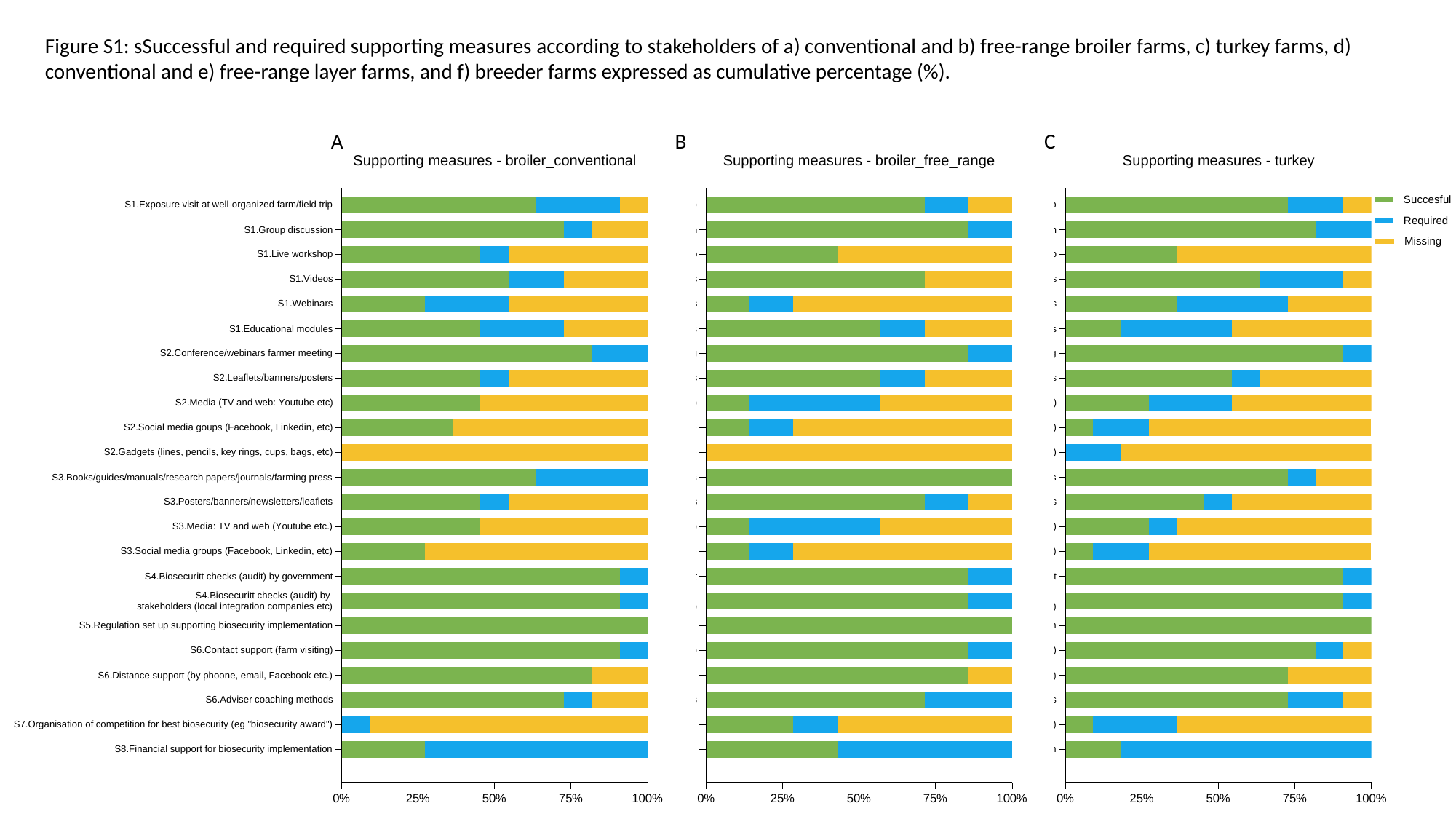

Figure S1: sSuccessful and required supporting measures according to stakeholders of a) conventional and b) free-range broiler farms, c) turkey farms, d) conventional and e) free-range layer farms, and f) breeder farms expressed as cumulative percentage (%).
C
B
A

## Slide 2
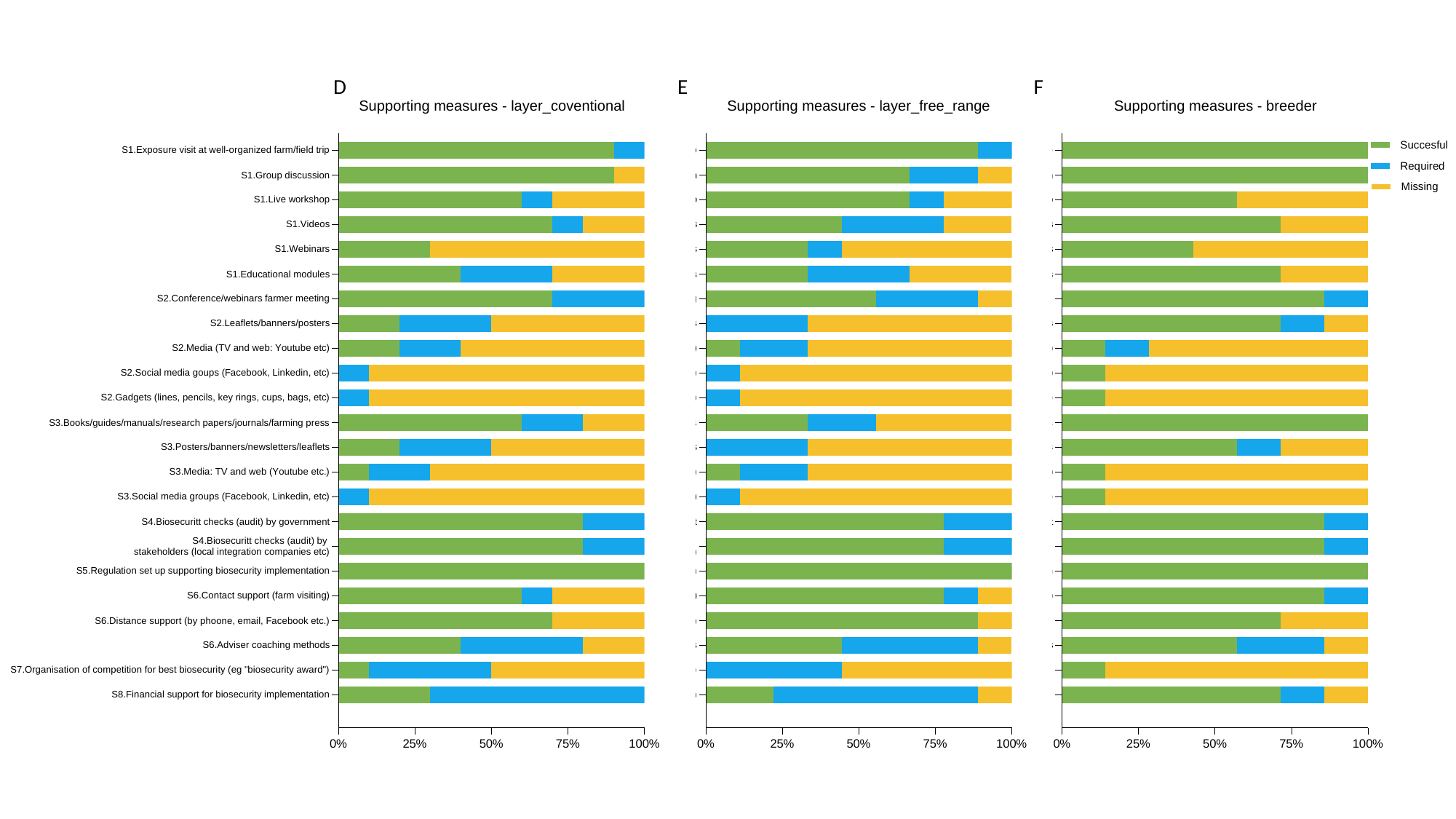

F
E
D
